# Decrease of Mutual Information in Brain Electrical Activity of Patients with Relapsing-Remitting Multiple Sclerosis

**DOI:** 10.3233/BEN-120278

**Published:** 2013

**Authors:** Bruno Lenne, Jean-Luc Blanc, Jean-Louis Nandrino, Philippe Gallois, Patrick Hautecæur, Laurent Pezard

**Affiliations:** ^1^Service de NeurologieHôpital Universitaire Saint-Philibert – GHICLLommeFrance; ^2^INSUMR 1106 InsermAix-Marseille UniversitéMarseilleFrance; ^3^URECA EA 1059Université de Lille Nord de FranceLilleFrance

**Keywords:** Multiple sclerosis, EEG signals, mutual information, coherence, inter-hemispheric communication

## Abstract

The disturbance of cortical communication has been hypothesized as an important factor in the appearance of cognitive impairment in (MS). Cortical communication is quantified here in control subjects and patients with relapsing-remitting multiple sclerosis (RRMS) on the basis of mean coherence in the *δ*, *θ*, *α*, *β* and *γ* bands and using mutual information computed between pairs of bipolar EEG signals recorded during resting condition. Each patient received also a cognitive assessment using a battery of neuropsychological tests specific to cognitive deficits in MS.

No difference was observed for the coherence indices whereas inter-hemispheric and right hemisphere mutual information is significantly lower in patients with MS than in control subjects. Moreover, inter-hemispheric mutual information decrease significantly with illness duration and right mutual information differentiate cognitively deficient and non-deficient patients.

Mutual information allows to quantify the cortical communication in patients with RRMS and is related to clinical characteristics.

Cortical communication quantified in a resting state might be a potential marker for the neurological damage induced by RRMS.

## 1. Introduction

Multiple sclerosis (MS) is an autoimmune disorder characterized by multiple lesions of the central myelin and accumulating clinical signs due to demyelination and progressive axonal damage [1]. As a consequence of white matter injury, partial or total cortical disconnection from subcortical and spinal targets occurs in parallel with deficits in cortico-cortical connectivity. Widespread demyelination and axonal damage in MS impairs the conduction of neural impulses which leads to physical and cognitive disability [2].

Cognitive impairment affects approximately 30 to 70% of MS patients and strongly affects their quality of life [3,4]. MS impairs several cognitive functions, including attention [5,6], information processing efficiency [5], executive functioning [7] and long term memory [8]. Cognitive functions that are supported by brain networks are thus highly dependent upon the integrity of long white matter tracts which mediate information flow between distant cortical areas [9]. Increased risk for cognitive decline in MS patients has been associated with the extent of diffuse tissue damage, lesion localisation, deficiency in neural connectivity and cortical reorganization [10]. Moreover, slower neural conduction induced by white matter pathology [11], grey matter pathology and cortical atrophy [12,13] and inter-hemispheric transfer perturbation due to callosal

ISSN 0953-4180/13/$27.50 © 2013 – IOS Press and the authors. All rights reserved lesions [14,15] have been successively considered as important factors for cognitive impairments in MS.

Damages of the corpus callosum (CC) in MS have been assessed by Magnetic Resonance Imaging (MRI) studies. Callosal lesions were found in more than 50% of patients and CC atrophy in more than 40% of patients [16]. These disturbances were even observed at the earliest stage of the disease [17]. Clinical evidence for functional impairment of callosal tracts were found in problem solving tasks, sustained attention or fast information processing [14,18–21]. Moreover, the reduction of interhemispheric crossings efficiency of auditivo-verbal and visual information has been correlated with the presence of CC atrophy [14]. Tasks which evaluate transfer of auditive, motor and somesthesic information also shown a deficit in interhemispheric transfer [22–24]. Some rare cases of MS patients presenting a real syndrome of callosal disconnection were also reported [25]. Ipsilateral Silent Period (ISP) paradigm revealed that transcallosal conduction is significantly slower in MS patients [26]. These studies underlined that demyelination which induces degeneration of conductive capacities, reduces communication between separate cortical regions and particularly between inter-hemispheric areas. These results have led to regard the morphological and functional evaluation of the CC as a diagnostic and prognostic index in disease follow-up [27].

These studies focussed on the spatial dimension of CC and its relationship with cognitive impairments in MS. Nevertheless, they neglect the direct assessment of callosal physiological efficiency. In a similar perspective, current efforts are devoted to delineating brain connectivity changes from an anatomical perspective through MRI techniques such as voxel-based morphometry and diffusion tensor imaging based fibre tracking [17,28–30]. However, such techniques mainly provide information on anatomical substrates related to pathways of large, unidirectional fibres and does not allow the investigation of rapid (millisecond range) phenomena linked with functional cortico-cortical connectivity such as transient increments/decrements of neural assembly activities. Electro-encephalography (EEG) represents a non-invasive neurophysiological technique with appropriate temporal resolution to detect such phenomena in humans. Previous studies of EEG coherence in progressive MS patients demonstrated a significant decrease of $\alpha$ and $\theta$-band coherence between both anteroposterior and interhemispheric areas which correlates with cognitive impairment and subcortical lesion load observed on MRI [31]. Likewise, a decreased $\alpha$-band interhemispheric coherence was found in relapsing-remitting MS (RRMS) patients through a MEG paradigm [32]. More recently, it has been suggested, using oscillatory steady-state responses to rhythmic auditory stimuli, that the loss of synchronization in different central nervous system pathways caused by demyelinating lesions might involve both the slowing of brain oscillatory activity and less efficient cognitive processing [33].

The goal of our study is to provide a global assessment of intra and interhemispheric communication using coherence and mutual information indices computed on electroencephalographic data obtained during the clinical assessment of MS. The cortical communication was investigated in control subjects and in patients suffering from RRMS. The cognitive functions of patients were assessed using a battery of neuropsychological tests (BCcogSEP, a french adaption of BRB-N, [34]). Multiple factor analysis was used to study the relationships between clinical data, neuropsychological assessments and the electrophysiological variables that discriminate between control subjects and MS patients.

## 2. Method

### *2.1. Participants*

The protocol was approved by the local medical committee and was included in the standard cares and follow-up of patients. All participants gave their written informed consent to participate to the study, after a thorough explanation of the procedure.

#### *2.1.1. MS patients*

Individuals were recruited during neurological consultation. This sample included 31 participants (see Table 2) with clinically defined RRMS, according to McDonald's criteria [35]. These patients were all diagnosed and treated at the Department of Neurology of GHICL Hospital (North of France). The mean years since diagnosis was 9.6 (SD = 7.1). Neurological status and disability were assessed by Expanded Disability Status Scale [36] which ranges from 0 (i.e. normal) to 10 (i.e. death). Patients' EDSS scores ranged from 1 to 5 (median: 3) which indicates a moderate mobility disability. All patients underwent a standardized clinical and cognitive assessment during a stable phase of the disease, at least one month after the most recent relapse and/or IV steroid course.

Table 1
Scores to neuropsychological tests (means and standard deviations) and proportions of deteriorated patients with the 95% confidence interval ($CI_{95}$) of this proportion [37]. In the last column, some cognitive score have been grouped for comparison with EEG data. Means of proportion of deteriorated patients for each group of cognitive variables are presented. PASAT: paced auditory serial task. SRT: selective reminding task. $CI_{95}$: 95% confidence interval

| Neuropsychological assessment | Mean | Standard deviation | % of patients with deterioration | $CI_{95}$ | % of patients with deterioration in each group of neuropsychological variable |
|---|---|---|---|---|---|
| SRT Recall | 10.92 | 2.16 | 38.71 | $CI_{95}$ [21.56; 55.86] | |
| SRT Learning index | 67.21 | 17.27 | 19.35 | $CI_{95}$ [5.44; 33.26] | 31.2% |
| SRT Delayed recall | 12.29 | 3.09 | 35.48 | $CI_{95}$ [18.64; 52.32] | $CI_{95}$ [14.89; 47.51] |
| 10/36 Recall | 16.13 | 5.77 | 19.35 | $CI_{95}$ [5.44; 33.26] | 27.42% |
| 10/36 Delayed recall | 5.87 | 2.58 | 35.48 | $CI_{95}$ [18.64; 52.32] | $CI_{95}$ [11.72; 43.12] |
| PASAT (3 sec.) | 60.17 | 18.68 | 51.61 | $CI_{95}$ [34.02; 69.2] | 50% |
| PASAT (2 sec.) | 46.89 | 17.50 | 49.39 | $CI_{95}$ [30.8; 65.98] | $CI_{95}$ [32.4; 67.6] |
| Code | 46.17 | 14.09 | 51.61 | $CI_{95}$ [34.02; 69.2] | 51.61% |
| | | | | | $CI_{95}$ [34.02; 69.2] |
| Digit span forward | 5.70 | 1.15 | 54.84 | $CI_{95}$ [37.32; 72.36] | – |
| Digit span backward | 4.73 | 1.26 | 58.06 | $CI_{95}$ [40.69; 75.43] | |
| Semantic fluency | 18.72 | 5.22 | 38.71 | $CI_{95}$ [21.56; 55.86] | 38.71% |
| Phonemic fluency | 13.55 | 4.68 | 38.71 | $CI_{95}$ [21.56; 55.86] | $CI_{95}$ [21.56; 55.86] |
| Crossed taping (errors) | 2.04 | 5.63 | 12.9 | $CI_{95}$ [1.1; 24.7] | – |
| Go/No Go (errors) | 1.19 | 3.16 | 9.68 | $CI_{95}$ [0.73; 20.09] | – |

Table 2
Demographic and clinical characteristics of MS patients and control subjects. Results of Fisher ($\chi^2$ statistics) and Wilcoxon ($W$ statistics) tests are given in the last column

| | Patients | Controls | |
|---|---|---|---|
| Number | 31 | 20 | |
| Sex (F:M) | 18:13 | 14:6 | $\chi^2 = 0.88$, n.s. |
| Mean Age, years (SD) | 39.5 (9.5) | 40.5 (11.5) | $W = 328$, n.s. |
| Mean scholar length, years (SD) | 14.3 (3.2) | 13.4(2.6) | $W = 451$, n.s. |
| Median EDSS (5–95 percentiles) | 3 (1-5) | – | |
| Mean disease duration, years (SD) | 9.6 (7.1) | – | |

### *2.1.2. Control subjects*

Control data for EEG parameters were obtained by recording a population of 20 healthy subjects (see Table 2). They were recruited through a community industrial medicine department and did not receive any retribution. Subjects with any neurological disease or known psychiatric illness, history of head trauma or alcohol or drug abuse were not included. These control subjects were matched with the patients for gender, age and educational level.

## *2.2. Clinical evaluation and neuropsychological assessment*

Patients completed clinical evaluation composed of both a battery of neuropsychological tests and of EEG recording performed by an experienced clinical neuropsychologist over two sessions of one hour spaced by at most one month without acute relapse.

The same psychologist conducted an extensive neuropsychological examination for each participant to identify cognitively deteriorated participants. No patient had ever had previous neuropsychological testing. The test battery (BCcogSEP) is built upon previously published neuropsychological findings [37] and is designed to assess a wide range of cognitive abilities. This battery based on the Brief Repeatable Battery for Neuropsychological examination [34] was used to assess several cognitive processes of patients, particularly those where the cognitive deficits are frequently observed in patients with MS. This battery consisted of eight tests with Selective Reminding Test [38, SRT] assessing learning capabilities and consolidating information in verbal modality, a test of visual-spatial learning [39], an adaptation of the Code subtest [40, WAIS-R], the Paced Auditory Serial Addition Task [41, PASAT], digit spans (direct and reverse), semantic and phonemic fluency (using respectively the category of animals and the letter “P”, as probes), the Crossed Tapping [42] and Go/No Go non-computerized. Fourteen scores were determined with this neuropsychological battery.

For each score, participants were classified as passing or failing the task whether their score falls in or outside the confidence interval of 95% ($CI_{95}$) of the control performance [37]. Using this cut-off, participants were considered as cognitively impaired if their score is lower than the fifth percentile of controls on at least four scores of the battery.

### 2.3. Electroencephalographic (EEG) recordings

EEG signals were recorded in Department of clinical neurophysiology (University Hospital of St-Philibert, Lomme, France) using 17 Ag/AgCl electrodes (C3, C4, Cz, F3, F4, F7, F8, Fz, O1, O2, P3, P4, T3, T4, T5, T6) placed on the scalp according to the 10/20 international electrode placement system. The reference electrode was placed on the nose. Prefrontal electrodes (Fp1, Fp2, Fpz) which are most likely to be contaminated by eye movements, were excluded from the analysis. EEG signals were digitized on 16 bits of precision using a 256 Hz sampling frequency and filtered using a band-pass filter between 0.5 Hz and 70 Hz. Twenty minutes of EEG recordings were obtained from each participant in eyes closed and resting condition.

### 2.4. EEG analysis

About 2 min. (i.e.: 117 sec. or $3.10^4$ time samples) of artifact-free EEG recordings were visually selected from each patient for the computation of coherence indices and mutual information.

Since we are mainly interested in the intra- and inter-hemispheric relationships, we first discarded the central electrodes (Fz, Cz) from our analysis. To avoid confounding inter-electrode coherence by the effect of common reference electrode [43,44] bipolar signals were used. This procedure produces a spatially filtered estimate of local activity which has been shown to exclude activity from more remote sources, including reference electrode [45,46]. This leads to the following ten bipolar recordings: T3-F7, T5-T3, C3-F3, P3-C3, O1-P3 for the left hemisphere and to C4-F4, P2-C4, O2-P4, T6-T4, T4-F8 for the right hemisphere. These derivations were obtained by substracting one measured potential from the other in each electrode pair prior to further analysis. Since all measurements matrices (coherence and mutual information) are square and symmetric, these 10 bipolar recordings lead to 45 relevant pairs of recordings where our indices are computed.

#### 2.4.1. Coherence

The coherence function is a classical measure of the linear synchronization between two time series $X = [x_n]$ and $Y = [y_n]$, $n = 1, \ldots, N$ (here $N \approx 3.10^4$) as a function of the frequency $\omega$ [47]. This measure is particularly useful when synchronization is limited to some particular frequency band. It is defined as the normalized cross-spectrum density:

$$C_{XY}(\omega) = \frac{|R_{XY}|^2(\omega)}{\sqrt{|R_{XX}|^2(\omega)}\sqrt{|R_{YY}|^2(\omega)}} \qquad (1)$$

where the cross spectrum of $X$ and $Y$: $|R_{XY}|^2 = \mathcal{F}[X]\overline{\mathcal{F}[Y]}$ (with $\mathcal{F}$ the Fourier transform and $\overline{\mathcal{F}[Y]}$ the conjugate of $\mathcal{F}[Y]$) corresponds to the Fourier transform of the linear cross-correlation function between $X$ and $Y$.

Normalized coherences $\langle C_{XY} \rangle$ were then obtained in the $\delta$, $\theta$, $\alpha$, $\beta$ and $\gamma$ bands as:

$$\langle C_{XY} \rangle_b = \frac{\sum_{\omega_{\min}<\omega<\omega_{\max}} C_{XY}(\omega)}{\sum_\omega C_{XY}} \qquad (2)$$

for $b$ in $\{\delta, \theta, \alpha, \beta, \gamma\}$, with $XY$ a pair of bipolar recordings and where $\omega_{\min} = 1$ Hz and $\omega_{\max} = 4$ Hz for the $\delta-$band, $\omega_{\min} = 4$ Hz and $\omega_{\max} = 8$ Hz for the $\theta-$band, $\omega_{\min} = 8$ Hz and $\omega_{\max} = 12$ Hz for the $\alpha-$band, $\omega_{\min} = 12$ Hz and $\omega_{\max} = 30$ Hz for the $\beta-$band and $\omega_{\min} = 30$ Hz and $\omega_{\max} = 50$ Hz for the $\gamma-$band. For each frequency band, there are 45 meaningful coefficients $\langle C_{XY} \rangle$ which were used as our coherence indices.

#### 2.4.2. Mutual information

Mutual information $I(X;Y)$ of two random variables $X$ and $Y$ reflects the reduction of uncertainty on one random variable when the other is known and thus measures the information transmission between $X$ and $Y$ [48]. It is a general measure of the strength of dependence between $X$ and $Y$. It vanishes for independent variables and it increases with the strength of the dependence [49].

Mutual information indices were computed between all the 45 pairs of bipolar channels using the following procedure:

1. The signal of each channel was transformed into an identically distributed process using $k = 30$ bins so that each symbol has the same probability of occurrence and entropy is maximal [50]. Namely, the interval between the minimum and the maximum value of X is divided into $k = 30$ sub-intervals (indexed from 0 to $k - 1$) each of which contain $1/30$ values of $X$. Bipolar signal $X = [x_n]$ (resp. $Y = [y_n]$) was thus transformed into a symbolic sequence $\mathcal{X} = [\xi_n]$ (resp. $\mathcal{Y} = [\upsilon_n]$) with $\xi_n$ (resp. $\upsilon_n$) belongs to $\Xi = \{0, 1, \ldots, k-1\}$ (resp. $\Upsilon = \{0, \ldots, k-1\}$) and $n = 1, \ldots, N$ where $\xi_n$ (resp. $\upsilon_n$) is the index of the interval where $x_n$ (resp. $y_n$) falls.

2. for each pair of symbolic representation of bipolar signals $\mathcal{X}$ and $\mathcal{Y}$, the mutual information $I(\mathcal{X};\mathcal{Y})$ was computed using:

$$I(\mathcal{X};\mathcal{Y}) = \sum_{\upsilon \in \Upsilon} \sum_{\xi \in \Xi} p(\xi, \upsilon) \log \left( \frac{p(\xi, \upsilon)}{p(\xi)p(\upsilon)} \right) \quad (3)$$

where $p(\xi, \upsilon)$ is the joint probability distribution of $\mathcal{X}$ and $\mathcal{Y}$ and $p(\xi)$ and $p(\upsilon)$ are the marginal probability distribution functions of $\mathcal{X}$ and $\mathcal{Y}$ respectively.

Since mutual information matrix is square and symmetric, we thus obtained 45 indices of mutual information for all the pairs of bipolar signals which allow us to characterize the nonlinear dependence between EEG signals.

### 2.5. *Statistical analysis*

#### 2.5.1. *Electrophysiological indices*

Electrophysiological indices (i.e. normalized coherences in each frequency band and mutual information indices) were analysed using a non-parametric cluster test [51–53]. For one single index, this test is composed of the following steps:

1. For each pair of bipolar signals, the average value of the index is computed for each group of subjects (i.e. for patients and for controls). The difference between these averaged values $\Delta_m$ is computed and corresponds to our experimental statistics ($m = 1, \ldots, 45$).
   A permutation test is then used to assess the significance of this experimental statistics. For this the null hypothesis is defined as the situation where there is no difference between groups. The distribution of $\Delta_m$ under the null hypothesis is then obtained by randomly assigning the values of the index to one of both groups and computing $\Delta_m$ for each permutation. The set of values of $\Delta_m$ obtained for a large number of permutations gives an estimates of the distribution of $\Delta_m$ under the null hypothesis. The experimental value of the statistics is considered as significant if it departs from the permutation distribution for a p-value lower than a threshold of $\alpha = 0.05$.
2. Based on the preceding step, a cluster-based statistics was determined. A cluster is defined as the set of pairs of bipolar signals with one common reference bipolar signal [52]. Each cluster was thus attributed a size (i.e. the number of pairs where the null hypothesis was rejected in step one) and a statistical weight (i.e. the difference between the threshold statistics and the experimental statistics observed in step one).
3. A second permutation test is then issued on the cluster-based statistics (i.e. size and weight). It allows to determine the distributions under the null hypothesis of absence of difference between the groups for the index of size and weight of the clusters. Only the clusters for which the cluster-based statistics is higher than that of the permutation distribution (with risk $\alpha = 0.05$) are selected as significant.

The permutations tests were performed using a procedure implemented by the author in the *Python* language [54,55]. Electrophysiological variables were selected when they differentiate patients and controls for both weight and size of the clusters.

#### 2.5.2. *Comparison between electrophysiological indices and neuropsychological scores*

Once significant clusters of pairs of bipolar signals were selected according to the procedure described above, we explored the comparison between clinical data which characterizes patients (i.e. age, score to EDSS scale and illness duration), scores to neuropsychological tests and electrophysiological indices (normalized coherences and mutual information). We thus face the problem of comparing three tables of data which can be solved using Multiple Factor Analysis [56, MFA][57]. MFA is a descriptive technique based on Principal Component Analysis (PCA) which uses weighted variables to take into account the presence of several heterogeneous measurements (here: clinical, neuropsychological and electrophysiological). The set of all variables are then projected into an optimal space in which the contribution of individuals and variables can be assessed such as the correlations between variables as in the PCA. This statistical analysis was performed using the *FactoMineR* package [58] for the *R* statistical programming language [59]

After this descriptive procedure, nonparametric correlations tests using Spearman correlation coefficient were used in order to define the main relationships between clinical, neuropsychological and EEG measurements.

Table 3
Statistical results for the links where both size and weight statistical criteria are significantly different between patients and controls. Mean mutal information (± standard deviation) is given for both groups together with the results of the $\Delta_m$ statistics of the permutation test. In all cases the p-values are lower than $10^{-4}$

| Links | Patients | Controls | Statistics |
|---|---|---|---|
| T3-F7 = P4-C4 | 0.061 ± 0.046 | 0.105 ± 0.069 | $\Delta_m = -$ 0.044 |
| T3-F7 = O2-P4 | 0.026 ± 0.014 | 0.042 ± 0.027 | $\Delta_m = -$ 0.016 |
| T3-F7 = T4-F8 | 0.048 ± 0.037 | 0.080 ± 0.049 | $\Delta_m = -$ 0.032 |
| T5-T3 = O1-P3 | 0.193 ± 0.131 | 0.307 ± 0.241 | $\Delta_m = -$ 0.114 |
| T5-T3 = T6-T4 | 0.045 ± 0.042 | 0.073 ± 0.058 | $\Delta_m = -$ 0.028 |
| C3-F3 = O2-P4 | 0.027 ± 0.014 | 0.040 ± 0.037 | $\Delta_m = -$ 0.013 |
| P3-C3 = O2-P4 | 0.064 ± 0.041 | 0.087 ± 0.055 | $\Delta_m = -$ 0.023 |
| P3-C3 = T4-F8 | 0.057 ± 0.042 | 0.088 ± 0.061 | $\Delta_m = -$ 0.031 |
| O1-P3 = P4-C4 | 0.076 ± 0.043 | 0.130 ± 0.090 | $\Delta_m = -$ 0.054 |
| O1-P3 = O2-P4 | 0.150 ± 0.089 | 0.204 ± 0.076 | $\Delta_m = -$ 0.054 |
| O1-P3 = T6-T4 | 0.062 ± 0.056 | 0.103 ± 0.059 | $\Delta_m = -$ 0.040 |
| O1-P3 = T4-F8 | 0.032 ± 0.023 | 0.048 ± 0.037 | $\Delta_m = -$ 0.016 |
| P4-C4 = O2-P4 | 0.105 ± 0.069 | 0.174 ± 0.133 | $\Delta_m = -$ 0.069 |
| P4-C4 = T6-T4 | 0.162 ± 0.085 | 0.225 ± 0.160 | $\Delta_m = -$ 0.063 |
| P4-C4 = T4-F8 | 0.124 ± 0.087 | 0.213 ± 0.180 | $\Delta_m = -$ 0.089 |
| O2-P4 = T6-T4 | 0.158 ± 0.139 | 0.284 ± 0.226 | $\Delta_m = -$ 0.126 |
| O2-P4 = T4-F8 | 0.041 ± 0.027 | 0.079 ± 0.099 | $\Delta_m = -$ 0.038 |
| T6-T4 = T4-F8 | 0.062 ± 0.050 | 0.140 ± 0.130 | $\Delta_m = -$ 0.077 |

## 3. Results

### 3.1. Electrophysiological data

The computation of cluster statistics were done using 10000 permutations and a statistical threshold equals to 0.05.

No significant cluster in any of the frequency-band has been characterized using the coherence indices.

For the mutual information index, 6 clusters were selected as significant based on the size statistic and 5 based on the weight statistic (see Fig. 1). In all those clusters, the mutual information index is significantly lower in the patient group than in the control group.

We can observe that 18 links where both criteria are fulfilled can be selected. The mutual information values obtained for these links are taken as our measurements that differentiate control subjects and patients. Within these links, 1 and 6 intrahemispheric links (out of the 10 possible links for each hemisphere) were selected for the left and the right hemisphere repectively whereas 11 interhemispheric links (out of the 25 possible links) were found as significant. These proportions only depict a tendency for difference (proportion test, $\chi^2 = 5.583$, $df = 2$, $p = 0.061$). The results are summed up in Table 3 and Fig. 2.

In order to keep the number of variables compatible with the number of subjects, we reduced, for further analysis, the number of electrophysiological variables to the three averaged values taken for the left, right and inter-hemispheric mutual information on the links where both criteria were fullfiled.

### 3.2. Neuropsychological assessments

#### 3.2.1. Description of the score to neuropsychological tests

The results to the neuropsychological tests are given in Table 1 where the proportions of cognitively deteriorated patients for each score of neuropsychological assessment are presented. Nearly one third of patients depict a deficit in verbal learning test, 28% in visual-spatial learning test. Half of patients had a slow speed of information processing (in the PASAT test or Code) and almost 39% of patients show a deficit in verbal fluencies. All together over 74% of patients had global cognitive deterioration in BCcogSEP, according to the criteria of [37], so 23 patients out of 31 had at least four scores below the fifth percentile of the performance of control subjects.

On the basis of these results and in order to analyze a set of variables adapted to the number of observed patients, we selected: the averaged scores to the Selective Reminding Test test, the Paced Auditory Serial Addition Test the 10–36 test the fluency test and the result to the Code test (and thus discarded the scores to digit span test, crossed taping test and Go/noGo). Those scores now represent our neuropsychological variables.

#### 3.2.2. Multivariate analysis

A multiple factor analysis was performed for the patient group to assess the relationships between the three groups of variables:

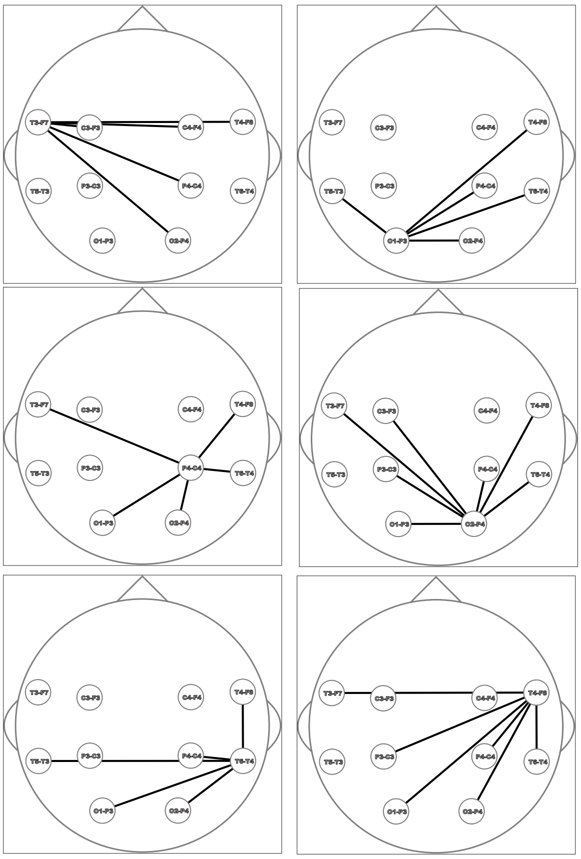

Fig. 1. Clusters selected using the size criterion. The clusters selected using the weight criterion are essentially the same except for the T3-F7 (top left) which is only selected on the basis of the size criterion. One can notice that the cluster with the smallest size (4) is discarded from the set of clusters on the basis of the weight criterion. Other clusters have sizes (or degrees) ranging from 5 to 7.

1. The clinical description variables (age, score to EDSS and disease duration)
2. The averaged scores to the neuropsychological tests (SRT, PASAT, 10/36, fluency test and Code)
3. The averaged left, right and interhemispheric EEG mutual information.

MFA leads to find a new basis for the multidimensional space that represents the data. The dimensions of this space are ordered by decreasing order of explained variance. Since the first clear decrease of percentage of explained variance appears between axis 3 and 4 (see Fig. 3), we restrict our analysis to the three first dimensions which explain a cumulative variance of 63.34% (23.05%, 18.94% and 18.35% for dimension 1, 2 and 3 respectively). The contribution of each variable to each dimension are given in Table 4. We observe that the first dimension is mainly defined by *Duree, EDSS, Code, pst and F*, the second one by *Age, Duree, EDSS, Inter* and the third one by *Age, inter, left, right*.

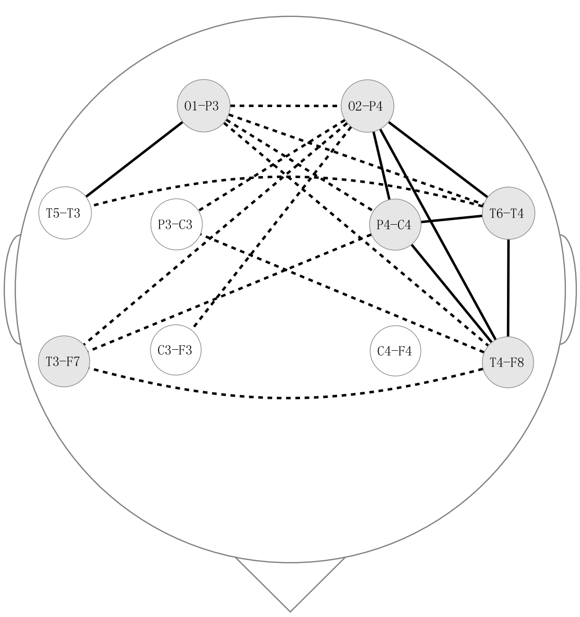

Fig. 2. Clusters and links where both criteria are fullfiled. Inter-hemispheric links are depicted with dashed lines, intra-hemispheric links for both right and left hemispheres are depicted with solid lines. Bipolar location where clusters are defined are filled in gray. We notice that two bilateral homologous areas appear: fronto-temporal (T3-F7 and T4-F8) and occipito-parietal (O1-P3 and O2-P4) and two right hemisphere areas: temporal (T6-T4) and parieto-central (P4-C4).

Table 4
Contribution of each variable to each dimension. The first three variables are clinical ones (age, duration and EDSS). The following three: “inter”, “left” and “right” refer to inter-hemispheric, left and right averaged mutual information respectively and the last five variables: “code”, “srt”, “pst” “ten36” and “F” refer to the score to the neuropsychological tests (code subtest, selective reminding test, 10/36 visuo-spatial learning and semantic and phonemic fluency test respectively)

| | Dim.1 | Dim.2 | Dim.3 |
|---|---|---|---|
| Age | 0.64 | 19.53 | 26.20 |
| Duree | 23.69 | 23.17 | 1.21 |
| EDSS | 16.73 | 22.09 | 2.30 |
| inter | 2.38 | 10.59 | 21.55 |
| left | 0.11 | 2.64 | 22.11 |
| right | 4.48 | 4.93 | 17.99 |
| Code | 13.82 | 3.74 | 3.35 |
| srt | 9.48 | 8.14 | 1.25 |
| pst | 12.17 | 0.27 | 1.27 |
| ten36 | 3.25 | 4.91 | 2.13 |
| F | 13.24 | 0.00 | 0.65 |

The MFA result in the projection of patients and variables in a reduced space with three dimensions. The distribution of patients in the planes (dim. 1 ×

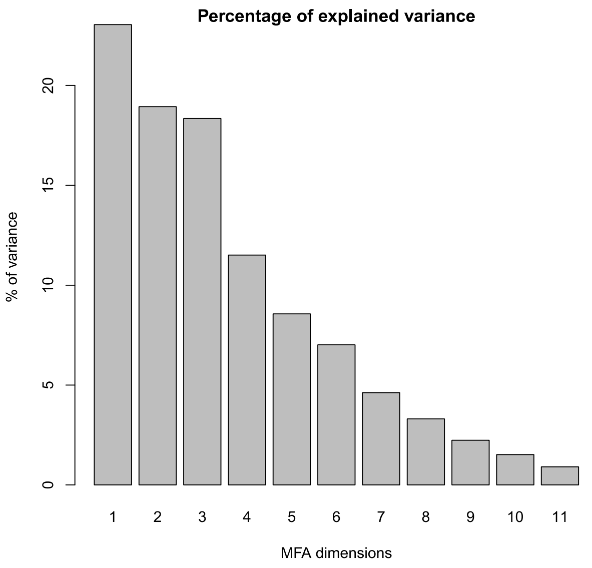

Fig. 3. Percentage of variance explained by the dimensions after Multiple Factor Analysis (MFA) of the three groups: clinical, neuropsychological and EEG variables.

dim. 2) and (dim. 1 × dim. 3) and the corresponding correlations circles are given in Fig. 4.

We can observe that although non-deficient patients (in red on the figures) are grouped, they cannot be completely separated from the deficient patients. The dimension 1 gives the best discrimination between these two groups of patients (Wilcoxon test: $W = 163$, $p < 0.001$) whereas dimension 2 and 3 do not discriminate these patients ($W = 79$, $p = 0.58$ and $W = 75$, $p = 0.46$ respectively). Nevertheless, this result is not surprising since the deficit is defined on neuropsychological scores and dimension 1 has a high contribution of neuropsychological variables. For electrophysiological measures, the averaged mutual information for the right hemisphere is significantly higher in the group of cognitively non-deficient patients (Wilcoxon test, $W = 30, p = 0.004$) whereas for the inter-hemispheric, one only observe a tendency (Wilcoxon test, $W = 50$, $p = 0.06$) and no difference for the left hemisphere (Wilcoxon test, $W = 68, p = 0.30$). These results are in accordance with the contribution of these variables to dimension 1.

The inspection of the correlation circles shows that neuropsychological scores and EEG mutual information are almost orthogonal (i.e. independant) variables and thus measures complementary dimensions of MS patients' characteristics. Moreover, neuropsychological scores are mainly negatively correlated with *EDSS* score (see Table 5) and EEG mutual information indices are mainly negatively correlated with *duration* of the illness (see Table 6). Only *Code* and EDSS and inter-hemispheric and duration of the illness depict a significant correlation.

Table 5
Correlations of neuropsychological variables (pst: paced auditory serial addition test, F: fluency test, code: code subtest, ten36: 10/36 visuo-spatial learning test and srt: selective reminding test) with EDSS. Correlation is quantified using Spearman's $\rho$ and tested rank statistics ($S$)

| | $\rho$ | $S$ | p-value |
|---|---|---|---|
| Code | −0.45 | 6515.13 | 0.01 |
| srt | −0.25 | 6190.77 | 0.18 |
| pst | −0.20 | 4888.54 | 0.29 |
| ten36 | −0.19 | 5886.25 | 0.31 |
| F | −0.23 | 4974.79 | 0.24 |

Table 6
Correlations of averaged EEG mutual information (inter-hemispheric, left and right hemisphere) with duration of the illness. Correlation is quantified using Spearman's $\rho$ and tested using rank statistics ($S$)

| | $\rho$ | $S$ | p-value |
|---|---|---|---|
| Inter | −0.35 | 6705.11 | 0.05 |
| Left | −0.05 | 5211.74 | 0.79 |
| Right | −0.22 | 6063.23 | 0.23 |

## 4. Discussion

In this study, functional information about inter- and intra-hemispheric cortical communication was obtained using standard EEG data obtained from patients with RMMS in resting eyes closed condition. The coherence in the $\delta$, $\theta$, $\alpha$, $\beta$ and $\gamma$-bands and mutual information computed between pairs of bipolar EEG signals were compared with those of control subjects. The electrophysiological indices that differentiated controls and patients were then compared with neuropsychological assessment and clinical characteristics of disease evolution using multiple factor analysis.

At odds with other previous studies, we did not find any difference between controls and patients for any EEG-band coherence. For example, a significant decrease of $\alpha$ and $\theta$ band coherence between both antero-posterior (in right and left hemisphere) and inter-hemispheric areas has been observed for patients with progressive multiple sclerosis which correlated with subcortical MRI lesion load [31]. However, since progressive form of MS occurs more lately, after RR evolution (for secondary progressive form) or in older patients (in secondary progressive and progressive forms) this decrease of coherence might be related to the age of the patient or the evolution of the disease. In fact, the patients of the present study are younger and de-

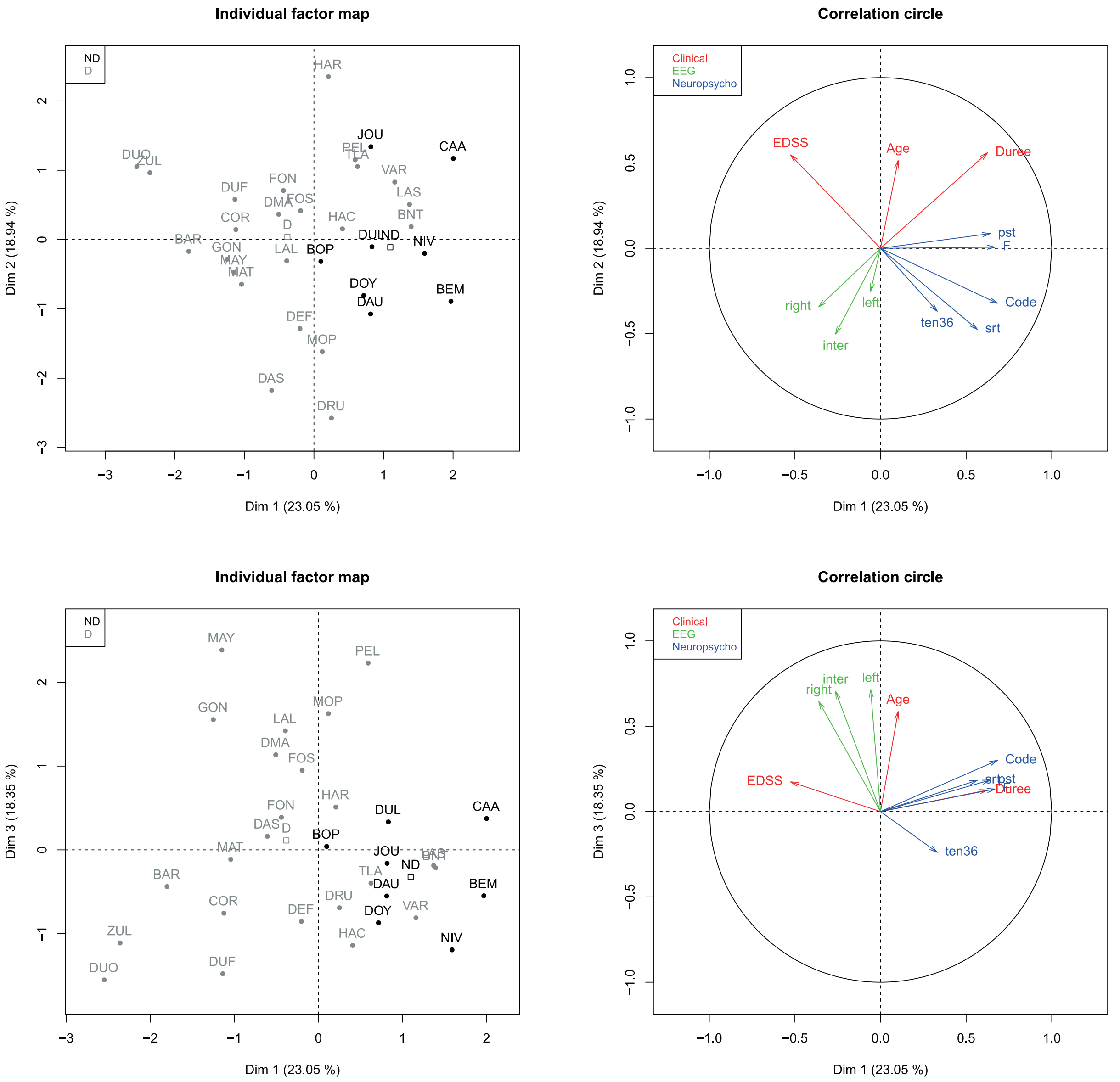

Fig. 4. Distribution of the individuals (left column) and correlation circles (right column) in planes: dim. 1 × dim. 2 (first line) and dim. 1 × dim. 3 (second line) after multiple factor analysis. On the left, the patient with cognitive deficiency (D) are shown in gray and those without cognitive deficiency (ND) are depicted in black. On the right, the clinical variables (age, duration of the illness and EDSS), the electrophysiological ones (EEG for inter-hemispheric, right and left averaged mutual information) and the neuropsychological variables (pst: paced auditory serial addition test, F: fluency test, code: code subtest, ten36: 10/36 visuo-spatial learning test and srt: selective reminding test) are shown in the correlation circle (resp. red, green and blue, in the online file: http://dx.doi.org/10.3233/BEN-120278).

pict a shorter evolution of the disease than those of this previous study. One can thus hypothesize that MRI lesion load could be more important in patients with progressive MS than in patients with relapsing-remitting MS such as those included in our study. These lesions might thus lead to more important EEG disturbance than those present in RRMS. In that case, coherence which mainly captures the linear part of the correlation between EEG signals would be less sensitive to EEG changes than mutual information which also captures the nonlinear part of EEG signals interdependences. Indeed, mutual information allowed us to differentiate cortical communication between RRMS patients and controls.

The main result of this study is the impairment of the cortical communication in RRMS characterized by a significant decrease of mutual information in a network of brain areas. This impaired network where the major decrease of mutual information was found is mainly composed by inter-hemispheric edges connected to a right hemisphere cluster of areas. This impairment contrasts with a relative preservation of left hemisphere communication. These results are partially in accordance with those obtained with coherence indices in patients with progressive MS [31] and can reflect the global disconnection of cortico-cortical or cortico-subcortical areas in MS. This network of decreased information transmission could be the physical support of a syndrome of intra- and interhemispheric disconnection [60,61] which damages both cortical and long myelinated fibers.

Our multiple factor analysis allowed us to characterize the relationship between the impairment of cortical communication implemented in the network of decreased mutual information and the clinical and neuropsychological characteristics of our group of RRMS patients. We first showed that the inter-hemispheric mutual information decreases significantly with the illness duration. Nevertheless, we did not find other correlation between EEG mutual information and cognitive deterioration or clinical scores such as EDSS. This result suggests that the decrease of inter-hemispheric communication worsen with disease evolution, but not with degree of disability. Our observation thus confirms the relative independence between disability and disease duration underlined in other study [62,63].

Our group of RRMS patients depicts a higher proportion of cognitively deteriorated patients (more than 74%) than usually reported in RRMS patients, e.g. near to 31% in [64] and 27% in [65]. Thus, the size of our group of non-deficient MS patients might be too small to allow clear statistical discrimination between cognitive performance, disease duration and degree of disability. Moreover, multiple factor analysis showed that the discrimination between groups of cognitively deficient and non-deficient patients is very difficult. Nevertheless, we found a lower mutual information in non-deficient patients than in deficient ones for the right hemisphere links of the impaired network. Since the brain electrical activity was not recorded during the task performance, this result is hardly interpretable on the basis of the present empirical data. Nevertheless, one can hypothesize that compensatory processes that allow non-deficient patients to perform cognitive tasks as normal subjects would change the balance of cortical information transmission when compared to deficient patients. The observed modification of right hemisphere mutual information should be a sign of this compensatory processes.

Since this study is focused on standard clinical EEG data obtained in a usual follow-up of patients with MS, we performed a global assessment of interhemispheric function during rest in RRMS patients. In such a non-specific experimental condition, mutual information was able to provide a quantification of interhemispheric communication in relation with disease course and a difference between cognitively deficient and non-deficient patients on the basis of right hemispheric cortical communication. It thus shows the putative clinical interest of this simple index. The study of mutual information of brain electrical activity during a task performance and the comparison with anatomical information such as MRI assessments of CC and periventricular atrophy are further complements of this study.

The present findings support that averaged interhemispheric mutual information obtained in a resting state is a marker for the neurological damage induced on patients with RRMS. Moreover these results suggest that such a non-linear measure could be an index of information processing deficit in MS patients. The clinical relevance of the present results should thus be tested in the follow-up of patients in longitudinal studies, with MRI data and more specific neuropsychological assessment. This simple non-linear measure of electrophysiological activity allows characterizing the connectivity of brain information transmission. It could also help characterize the evolution of the cortico-cortical and cortico-subcortical connection impairment, in addition to the characterization of interhemispheric transfer.

## Acknowledgment

We are indebted to J. Martinerie for pointing us to the non-parametric cluster statistics. This study was supported by the NeuroInformatics Interdisciplinary program of the CNRS under the CODYSEP acronym.
